# Negative feedback loop of ERK/CREB/miR‐212‐3p inhibits HBeAg‐induced macrophage activation

**DOI:** 10.1111/jcmm.15723

**Published:** 2020-08-07

**Authors:** Wenjun Chen, Hongjun Bian, Xiaoyu Xie, Xia Yang, Benjun Bi, Chunliu Li, Yuejuan Zhang, Qiang Zhu, Jing Song, Chengyong Qin, Jianni Qi

**Affiliations:** ^1^ Shandong Provincial Hospital Cheeloo College of Medicine Shandong University Jinan China; ^2^ Shandong Provincial Hospital Affiliated to Shandong First Medical University Jinan China; ^3^ The Affiliated Weihai Second Municipal Hospital of Qingdao University Weihai China; ^4^ Shandong Provincial Engineering and Technological Research Center for Liver Diseases Prevention and Control Jinan China; ^5^ The Affiliated Hospital of Qingdao University Qingdao China; ^6^ Yantai Affiliated Hospital of Binzhou Medical University Yantai China

**Keywords:** CREB, HBeAg, macrophage, MAPK1, miR‐212‐3p

## Abstract

The activation of liver macrophages is closely related to liver injury after HBV infection. Our previous results demonstrated that HBeAg played a key role in inducing macrophage activation. As we all know, miRNAs are involved in the regulation of multiple immune cell functions. Meanwhile, we have shown that miR‐155 positively regulates HBeAg‐induced macrophage activation and accelerates liver injury. Subsequently, based on our previous miRNA sequencing results, we further evaluated the role of miR‐212‐3p called ‘neurimmiR’ in HBeAg‐induced macrophages in this study. First, miR‐212‐3p expression was significantly elevated in HBeAg‐treated macrophages. Meanwhile, we found up‐regulation of miR‐212‐3p significantly decreased the production of cytokines, whereas knockdown of miR‐212‐3p held the opposite effect by gains and losses of function. Mechanically, although MAPK signal pathway, including ERK, JNK and p38, was activated in HBeAg‐induced macrophages, only ERK promoted the expression of miR‐212‐3p via transcription factor CREB, which was able to bind to the promoter of miR‐212‐3p verified by ChIP assay. Moreover, we further indicated that up‐regulated miR‐212‐3p inhibited HBeAg‐induced inflammatory cytokine production through targeting MAPK1. In conclusion, miR‐212‐3p was augmented in HBeAg‐stimulated macrophages via ERK/CREB signal pathway and the elevated miR‐212‐3p suppressed inflammatory cytokine production induced by HBeAg through targeting MAPK1.

## 1 INTRODUCTION

Human hepatitis B virus (HBV) is an international public health problem, and more than 250 million people are livingly infected with HBV all over the world. HBV infection can cause both acute and chronic liver damage. The immune response after HBV infection is essential to controlling HBV replication, but it also brings inflammatory damage to liver inevitably.^1^ It is well known that HBV can not only replicate in hepatocytes but also encode a variety of viral proteins such as HBsAg, HBeAg, HBcAg and HBX protein after they infect hepatocytes. Clinically, we often judge HBV infection status by detecting the content of these proteins in patients' peripheral blood. However, the role and mechanism of HBV‐associated proteins, including HBcAg, HBeAg and HBsAg, in affecting immune cells remain incompletely characterized.

Macrophages, a mature form of monocytes, participate in both innate immunity and adaptive immunity. They usually are the first defence in innate immunity.^2^ In the case of bacteria infection, the molecules such as LPS, peptidoglycan (PGN) and lipopeptides activate macrophages via specific receptors like TLRs. Upon activation, cytokines and chemokines are released via NF‐κB or other key transcription factors binding to their promoter, which cause quick inflammatory response. Multiple evidences showed that macrophages were involved in the process of HBV‐associated immune‐mediated liver injury.^3^, ^4^, ^5^ However, the effect of HBV‐associated proteins on macrophage activation and the subsequent liver damage remains unclarified. According to our previous findings, HBeAg, but not HBcAg and HBsAg, played a key role in inducing macrophage activation and exacerbated liver injury by increasing the production of inflammatory cytokines.^6^ Even so, the mechanism of HBeAg‐induced macrophage activation is not clear.

MiRNAs, about 18‐25 nucleotides length, are non‐coding RNA molecules, and they can regulate the gene expression at post‐transcriptional level via interaction with the 3′ untranslated regions (UTR) of their target mRNAs.^7^, ^8^ It has been verified that miRNAs were closely correlated with multiple pathological processes, such as tumour progression,^9^ neurological diseases,^10^ inflammatory diseases, cardiovascular diseases and liver diseases.^11^ Recently, a number of miRNAs have been identified to regulate inflammatory response. For example, miR‐210 was reported to inhibit LPS‐induced inflammation via targeting NF‐κB1 in murine macrophages,^12^ miR‐93 can suppress the production of inflammatory cytokine in LPS‐induced murine macrophages via IRAK4,^13^ and miR‐147 was demonstrated to negatively regulate the inflammatory response of macrophage.^14^ While miR‐155 was revealed to promote inflammatory response and aggravate liver injury by targeting BCL‐6, SHIP‐1 and SOCS‐1,^6^ miR‐101 was shown to modulate the innate immune responses of macrophages by targeting MAPK phosphatase‐1.^15^ Previously, miR‐212 has been widely reported to be involved in neurological diseases^16^, ^17^ and tumours,^18^ but there are not many reports about miR‐212 and inflammation especially in HBV infection.

In our study, we firstly found that miR‐212‐3p was up‐regulated in HBeAg‐induced macrophages. Next, we identified that ERK/CREB signalling pathway promoted the expression of miR‐212‐3p. In the end, our data indicate that miR‐212‐3p functioned to inhibit HBeAg‐induced inflammatory cytokine production in macrophages by targeting MAPK1. This study, to our knowledge, is the first to investigate the expression and function of miR‐212‐3p in HBeAg‐induced macrophages.

## 2 MATERIALS AND METHODS

### 2.1 Cell lines and reagents

The mouse macrophage cell line RAW264.7 (American Type Culture Collection, Manassas, VA, USA) was maintained as described previously.^6^ Human monocytic cell line THP‐1 (Procell Life Science, Wuhan, China) was cultured in RPMI‐1640 + 10% FBS + 0.05 mmol/L β‐mercaptoethanol + 1% P/S. Human leukaemia cells U937 were grown in RPMI‐1640 culture medium containing 10% (vol/vol) FBS (Thermo Fisher Scientific, Waltham, MA, USA). All cell lines were stored at 37°C in a humidified incubator with 5% CO_2_. HBeAg (ab91273) and anti‐CREB (phospho S133) antibody (ab32096) were purchased from Abcam (Cambridge, MA, USA). Rabbit mAb to GAPDH (10494‐1‐AP) was obtained from Proteintech Group Rosemont, IL, USA, Inc Antibodies for ERK (#4695), p‐ERK (#9101), JNK (#9252), p‐JNK (#9251), P38 (#9212), p‐P38 (#9211) and CREB (48H2) were all acquired from Cell‐Signaling (Cell‐Signaling Technology, Boston, MA, USA). The corresponding HRP‐conjugated secondary antibodies were acquired from Proteintech Group, Inc PD98059 (ERK inhibitor, HY‐12028), SP600125 (JNK inhibitor, HY‐12041) and SB203580 (p38 inhibitor, HY‐10256A) were purchased from MCE (MedChemExpress, Pudong New Area, Shanghai, China). KG‐501(CREB inhibitor, S8409) was purchased from Selleck (Selleck Chemicals, Houston, TX, USA). The study was approved by the ethics committee of Shandong Provincial Hospital Affiliated to Shandong University.

### 2.2 Isolation of human peripheral blood monocytes

10 healthy controls and 20 patients with CHB (chronic hepatitis B) were enrolled from Shandong Provincial Hospital, Cheeloo College of Medicine, Shandong University. The criteria established the National Viral Hepatitis Conference of China (2015) were used to diagnosis CHB. The examination result of antibody to HAV, HCV, HDV, HEV and HIV of them was all negative. We isolated human peripheral blood monocytes (PBMs) according to our previously study.^6^, ^7^ Brief description is as follows: PBMs were acquired with the method of density gradient centrifugation in 780 *g* for 25 minutes at room temperature and seeded in a 12‑well plate with RPMI‑1640 medium (Gibco^®^; Thermo Fisher Scientific Waltham, MA, USA, Inc) supplemented with 10% FBS to attach for overnight. The next day, the floating suspended cells were removed and the remaining adherent cells were rinsed twice with RPMI‑1640 medium. And then, they were incubated with or without HBeAg for 24 hours. These cells were collected for subsequent experiments.

### 2.3 Cells transfection

MiR‐212‐3p mimics and inhibitor and their corresponding negative control (NC) were purchased from GenePharma Corporation (Shanghai, China). HiPerFect Transfection Reagent (301705; Qiagen, Dusseldorf, Germany) was used for transfection. Briefly, after cells were seeded in the wells, we mixed the HiPerFect Transfection Reagent and miR‐212‐3p mimics or inhibitor or negative control according to manufacturer's instructions. The cells were incubated in medium without serum with transfected complexes for 6 hours, and then, medium containing serum was added to the cells. RNA or protein was extracted from cells for further study after transfection for 48‐72 hours.

### 2.4 RNA extraction and q‐PCR for mRNA and miRNA quantification

Total RNA was extracted using TRIzol reagent according to the manufacturer's protocol (TaKaRa, Kusatsu, Shiga, Japan). For the quantification of miR‐212‐3p, cDNA was obtained with the Mir‐X miRNA First‐Strand Synthesis Kit. The cDNA templates were analysed using corresponding primer pairs and SYBR qRT‐PCR kit (TaKaRa) according to manufacturer's instructions. For other mRNA quantification, the cDNA synthesis was performed with a First‐Strand cDNA Synthesis kit (RR047A; TaKaRa). Expression of IL‐6, TNF‐α was determined by qRT‐PCR with SYBR Premix Ex Tap™ (TaKaRa). The relative expression level of miR‐212‐3p and mRNA were normalized against U6 snRNA and GAPDH respectively. The specific primers were as follows: 5′‐GCCTT CTTGG GACTG ATGCT‐3′ (sense) and 5′‐GCCAT TGCAC AACTC TTTTC TCA‐3′ (antisense) for IL‐6; 5′‐CGGGC AGGTC TACTT TGGAG‐3′ (sense) and 5′‐ACCCT GAGCC ATAAT CCCCT‐3′ (antisense) for TNF‐α and 5′‐AGGTC GGTGT GAACG GATTT G‐3′ (sense) and 5′‐TGTAGACCATGTAGTTGAGGTCA‐3′ (antisense) for GAPDH; 5′‐GGAAC GATAC AGAGA AGATT AGC‐3′ (sense) and 5′‐TGGAA CGCTT CACGA ATTTG CG‐3′ (antisense) for U6; 5′‐UAACA GUCUC CAGUC ACGGC CA‐3′ (sense) and 5′‐GCCGU GACUG GAGAC UGUUA UU‐3′ (antisense) for mmu‐miR‐212‐3p mimics; 5′‐UGGCC GUGAC UGGAG ACUGU UA‐3′ for mmu‐miR‐212‐3p inhibitor; 5′‐UUCUC CGAAC GUGUC ACGUT T‐3′ (sense) and 5′‐ACGUG ACACG UUCGG AGAAT T‐3′ (antisense) for negative control; 5′‐CAGUA CUUUU GUGUA GUACA A‐3′ for inhibitor negative control. The LightCycler Real‐time PCR System (Roche Diagnostics, Indianapolis, IN, USA) was used to perform the real‐time PCR analyses.

### 2.5 Enzyme‑linked immunosorbent assay (ELISA)

We collected the cell‐culture supernatants, and the concentrations of TNF‐α (KMC3011) and IL‐6 (KMC0061) were detected using the commercially available ELISA kits (Invitrogen, Carlsbad, CA, USA). All steps were performed according to the instructions of manufacturer.

### 2.6 Western blot

After washed with cold phosphate‐buffered saline (PBS) for two times, the cultured cells were lysed with RIPA lysis buffer (Beyotime Biotechnology, Shanghai, China) supplemented with a cocktail of protease inhibitors. The concentrations of protein were measured using the bicinchoninic acid assay (Pierce, Appleton, WI, USA). Equivalent amounts of proteins, approximately 30 μg, were separated in 10% SDS/PAGE and electro‐transferred onto PVDF membranes (Millipore, MA, USA). The membranes were blocked with 5% non‐fat milk for 2 hours at room temperature and then were incubated with the primary antibodies overnight at 4°C on a shaking table. After washed with TBST for three times, the membranes were incubated with the secondary antibody (Proteintech Group). The last analysis was conducted using ECL reagent kit (Millipore) at room temperature. GAPDH was used as internal loading control.

### 2.7 Chromatin immunoprecipitation assay (ChIP)

ChIP assay was performed using the EZ‐Magna ChIP kit (Millipore, Merck KGaA, Darmstadt, Germany). HBeAg‐stimulated and non‐stimulated RAW264.7 cells were cultured to approximately 1 × 10^7^, proteins and nucleic acids were cross‐linked by 1% formaldehyde at room temperature. Cells were lysed and treated by ultrasound to obtain appropriate sized pieces of DNA. Protein‐DNA complexes were immunoprecipitated with anti‐CREB antibody. After being eluted and reverse cross‐linked, the purified DNA was obtained by centrifugal column. The DNA was then assessed by standard end‐point PCR. The sequences of the primers utilized to amplify a 300 bp segment of CREB binding to the miR‐212 promoter were as follows: forward, 5′‐TTCCC TGTCC CGTCC CTTCC‐3′, reverse, 5′‐TATCC CGTCG CCCGC AGTT‐3′. The PCR products were subjected to agarose gel electrophoresis.

### 2.8 Statistical analyses

All data were presented as the mean ± standard deviation (SD). GraphPad Prism 7 software was used for analysing the data. Statistical significance was calculated by unpaired two‐tailed *t* test, and *P*‐values are classified as follows: *P < 0.05; **P < 0.01; ***P < 0.001; and *P* < 0.05 are considered as significant.

## 3 RESULTS

### 3.1 HBeAg promotes the expression of miR‐212‐3p in macrophages

**FIGURE 1:**
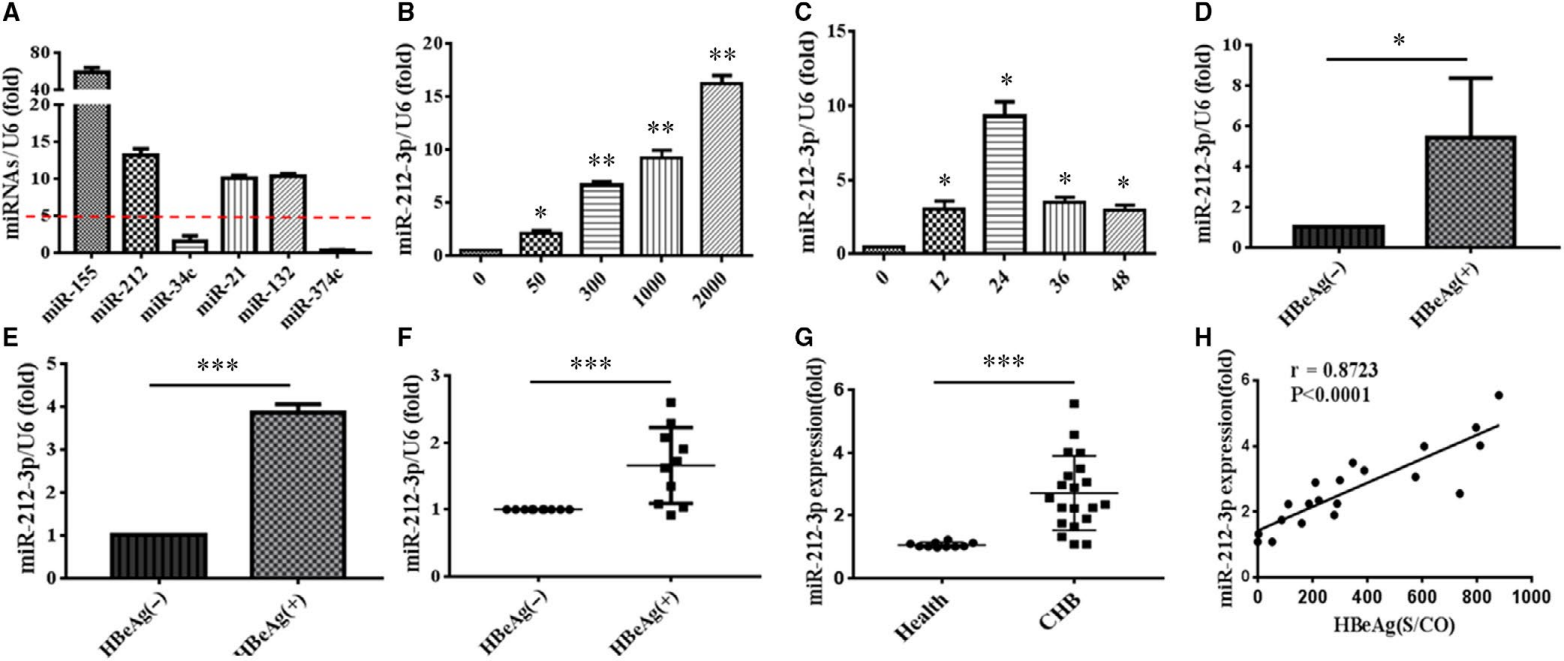
Expression of miR‐212‐3p was augmented in macrophages stimulated by HBeAg. RAW264.7 macrophages were stimulated by HBeAg (2000 ng/mL) for 24 h, and then, the expression of miR‐155, miR‐212‐3p, miR‐34c, miR‐21, miR‐132 and miR‐374c was assessed with q‐PCR (A). RAW264.7 macrophages were stimulated with different doses of HBeAg (0, 50, 300, 1000, 2000 ng/mL) for 24 h; the expression of miR‐212‐3p was tested by q‐PCR (B). RAW264.7 macrophages were treated with HBeAg (1000 ng/mL) for different time as shown; the expression of miR‐212‐3p was analysed by q‐PCR (C). THP‐1 cells were stimulated with HBeAg (2000 ng/mL) for 24 h; the level of miR‐212‐3p was detected by q‐PCR (D). U937 cells were stimulated with HBeAg (2000 ng/mL) for 24 h; the level of miR‐212‐3p was examined by q‐PCR (E). Peripheral blood monocytes from 10 normal subjects were treated with HBeAg (2000 ng/mL) for 24 h, respectively; the level of miR‐212‐3p was detected by q‐PCR (F). The peripheral blood monocytes of 20 patients with CHB were isolated, and the expression of miR‐212‐3p was detected by q‐PCR (G). The correlation between miR‐212‐3p expression and the content of HBeAg in 20 patients with CHB was analysed (H). Data shown above are representative of at least three independent experiments (mean ± SD of triplicates in A‐G). *P < 0.05, **P < 0.01, ***P < 0.001

According to the result of miRNAs sequencing performed previously, we performed q‐PCR assay and found that a series of miRNA was significantly elevated in RAW264.7 macrophages stimulated by HBeAg for 24 hours (Figure 1A). Previously, researchers from our laboratory have verified that miR‐155 is involved in this process and promotes production of inflammatory cytokines in macrophages.^6^ Next, we wanted to verify the role of the other elevated miRNAs except for miR‐155 during this process. We found that the level of miR‐212‐3p was significantly elevated and in a dose‐dependent manner (Figure 1B) in HBeAg‐stimulated macrophages. When we treated macrophages with HBeAg for various times, we found that the level of miR‐212‐3p was time‐dependent and reached its peak at 24 hours (Figure 1C). Apart from RAW264.7, we stimulated human THP‐1 (Figure 1D) and U937 (Figure 1E) cell lines with HBeAg for 24 hours and the results showed that the expression of miR‐212‐3p also increased significantly in the above cell lines after stimulation. In order to understand the clinical significance of miR‐212‐3p in human, we stimulated normal human PBMs with HBeAg. Interestingly, the level of miR‐212‐3p was also augmented in normal human peripheral blood monocytes after stimulation with HBeAg for 24 hours (Figure 1F). In addition, we isolated the PBMs of 20 patients with CHB and detected the expression of miR‐212‐3p. As shown in Figure 1G, there was a significant increase of miR‐212‐3p expression in PBMs of CHB patients compared with that of healthy controls. Moreover, the expression of miR‐212‐3p was positively correlated with their HBeAg content in CHB patients (Figure 1H). All together, we speculate that miR‐212‐3p may play a role in regulating inflammatory processes of macrophages induced by HBeAg.

### 3.2 miR‐212‐3p inhibits HBeAg‐induced inflammatory cytokine production in macrophages

**FIGURE 2:**
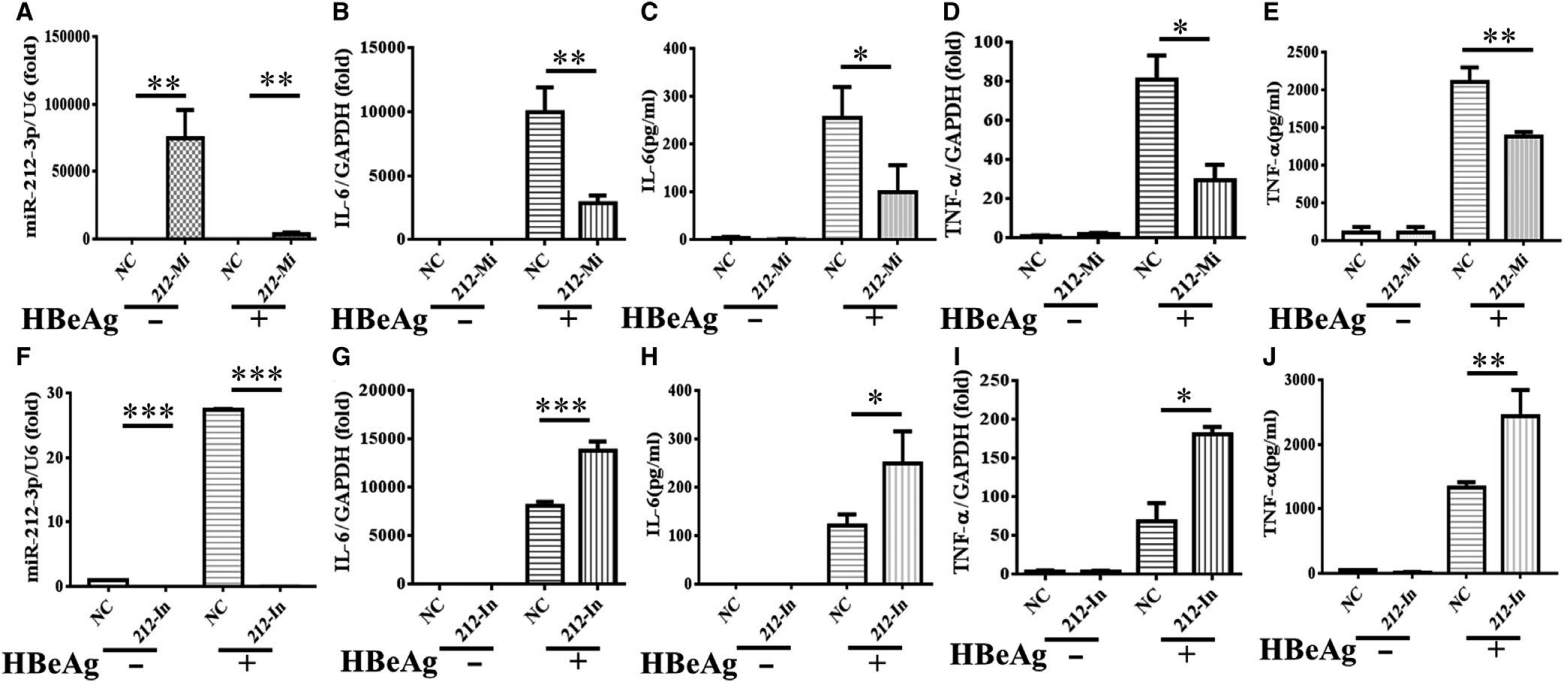
The inflammatory response of HBeAg‐induced macrophages was inhibited by miR‐212‐3p. miR‐212‐3p mimics and negative control were transfected into RAW264.7 macrophages for 24 h, then the cells were treated with HBeAg for 24 h, and the expression of miR‐212‐3p (A) and level of IL‐6 (B, C), TNF‐α (D, E) were tested by q‐PCR and ELISA. miR‐212‐3p inhibitor and negative control were transfected into RAW264.7 macrophages for 24 h, then the cells were treated with HBeAg for 24 h; and the expression of miR‐212‐3p (F) and level of IL‐6 (G, H), TNF‐α (I, J) were tested by q‐PCR and ELISA. Data are shown in at least three independent experiments (mean ± SD of triplicates in A‐J). *P < 0.05, **P < 0.01, ***P < 0.001

In order to explore the biological function of miR‐212‐3p in HBeAg‐induced macrophage inflammatory process, we performed the transfection experiment lead to the overexpression or knockdown of miR‐212‐3p in RAW264.7. First, the gain or loss efficiency was evaluated by qRT‐PCR (Figure 2A,F). From the results, we know that the expression of miR‐212‐3p is significantly up‐regulated after transfected by miR‐212‐3p mimics and is obviously down‐regulated after transfected by miR‐212‐3p inhibitor, compared with their corresponding controls. Next, the two pivotal cytokines during inflammatory process including IL‐6 and TNF‐α were assessed by qRT‐PCR and ELISA at mRNA and protein level, respectively. As shown in Figure 2B‐E, up‐regulation of miR‐212‐3p by miR‐212‐3p mimics significantly decreased the production of IL‐6 and TNF‐α at both mRNA and protein level after being treated with HBeAg in RAW264.7. On the contrary, knockdown of miR‐212‐3p by miR‐212‐3p inhibitor dramatically increased the production of IL‐6 and TNF‐α at both mRNA and protein level after being treated with HBeAg, which can be shown in Figure 2G‐J. Taken together, our results show that miR‐212‐3p negatively regulate inflammatory response of HBeAg‐induced macrophages by inhibiting the production of inflammatory cytokines.

### 3.3 HBeAg may activate the MAPK pathway which is involved in the regulation of macrophages activation

**FIGURE 3:**
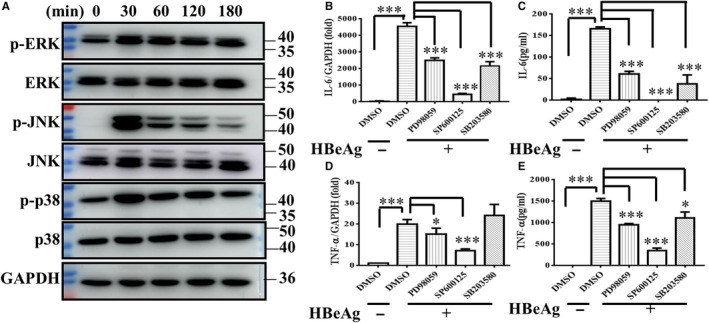
MAPK pathway was activated in HBeAg‐stimulating macrophages and mediated the production of inflammatory cytokines. (A) After RAW264.7 macrophages treated with HBeAg for various times (0, 30, 60, 120, 180 min), the expression of non‐ phosphorylation and phosphorylation of ERK, JNK and p38 was detected by Western blot. RAW264.7 macrophages were pre‐treated with DMSO or inhibitor of ERK(PD98059 20 μmol/L), JNK (SP600125 20 μmol/L) and P38(SB203580 20 μmol/L) for 1 h, respectively, and then stimulated with HBeAg for 4 h, and the expression and production of IL‐6 (B, C) and TNF‐α (D, E) were examined with q‐PCR and ELISA. Data are representative of three or four independent experiments (mean ± SD of triplicates in B‐E). *P < 0.05, ***P < 0.001

MAPK pathway plays a critical role in the process of innate immune responses. Previous studies demonstrated that LPS stimulation could activate MAPK signalling and lead to production of cytokines, including IL‐6 and TNF‐α in macrophages.^19^ To explore the role of MAPK in macrophage activation induced by HBeAg, we performed Western blot experiment to detect the expression of non‐ phosphorylation and phosphorylation of MAPK in RAW264.7 cells treated with HBeAg. As can be seen in Figure 3A, the phosphorylated levels of key proteins in MAPK signal pathway, including ERK, JNK and p38, were all elevated after treatment with HBeAg and reached their peak at 30 minutes, and then began to decrease gradually at 60 minutes. Moreover, in Figure 3B‐E, to further explore the role of MAPK pathway, we pre‐treated RAW264.7 cells with inhibitors of ERK, JNK and p38, respectively, and the production of IL‐6 and TNF‐α at both mRNA and protein level after being treated with HBeAg was all suppressed significantly. Taking all the above into consideration, we infer that MAPK including ERK, JNK and p38 were all activated and they promoted the activation of HBeAg‐induced macrophages via modulating cytokines production.

### 3.4 ERK/CREB signalling pathway promotes the expression of miR‐212‐3p

**FIGURE 4:**
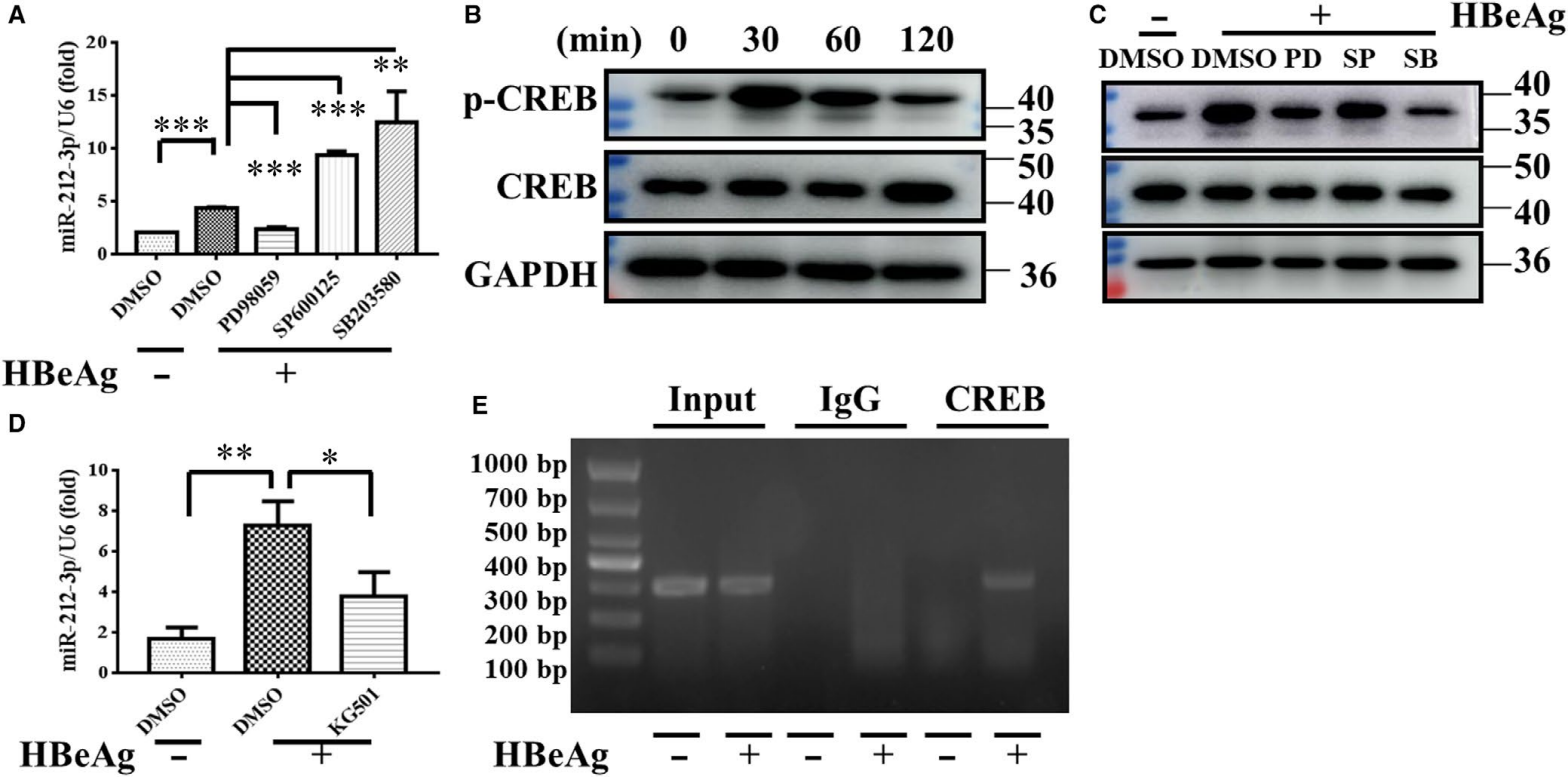
ERK/CREB signalling regulated the expression of miR‐212‐3p in HBeAg‐induced macrophages. After pre‐treated with DMSO or inhibitor of ERK (PD98059 20 μmol/L), JNK (SP600125 20 μmol/L) and p38 (SB203580 20 μmol/L) for 1 h, RAW264.7 macrophages were stimulated with HBeAg for 24 h, and then, the level of miR‐212‐3p was evaluated with q‐PCR (A). The expression of non‐ phosphorylation and phosphorylation of CREB in RAW264.7 macrophages was analysed with Western blot after stimulated by HBeAg for different time (0, 30, 60, 120 min) (B). RAW264.7 macrophages were pre‐treated with DMSO or inhibitor of ERK (PD98059, 10 μmol/L), JNK (SP600125, 10 μmol/L) and p38 (SB203580, 10 μmol/L) for 1 h and then were treated with HBeAg for 30 min, the level of non‐ phosphorylation and phosphorylation of CREB was measured by Western blot (C). After pre‐treated with DMSO or inhibitor of CREB (KG‐501, 10 μmol/L) for 1 h, RAW264.7 macrophages were stimulated with HBeAg for 24 h. q‐PCR analysed the level of miR‐212‐3p (D). RAW264.7 macrophages nuclear lysates were immunoprecipitated by anti‐CREB antibody or non‐specific IgG antibody. The antibody‐bound DNA sequences were assessed by standard end‐point PCR and agarose gel electrophoresis. This graph shows the binding of CREB with the miR‐212‐3p proximal promoter with or without stimulation by HBeAg (E). Similar results were obtained in three independent experiments (mean ± SD of triplicates in A, D). *P < 0.05, **P < 0.01, ***P < 0.001

Given the important function of miR‐212‐3p and MAPK in macrophage activation, we further investigated the relationship between MAPK and miR‐212‐3p. We pre‐treated RAW264.7 cells with DMSO or inhibitors of ERK, JNK and p38, respectively, and then stimulated RAW264.7 cells with HBeAg for 24 hours and detected the expression of miR‐212‐3p. From Figure 4A, we found that the increased expression of miR‐212‐3p induced by HBeAg is only significantly inhibited by ERK inhibitor. Therefore, we speculated that ERK can regulate the expression of miR‐212‐3p. As we all know, CREB is an important transcription factor located downstream of MAPK and regulates multiple signalling pathways. In addition, we found that there are many binding sites of CREB in miR‐212 promoter region by Jaspar software. Firstly, to clarify the role of CREB in macrophage activation, we treated RAW264.7 with HBeAg at different time‐points such as 0, 30, 60 and 120 minutes, and then detected the expression of non‐ phosphorylation and phosphorylation of CREB by Western blot. It can be seen in Figure 4B that the expression level of phosphorylated CREB increased significantly at 30 minutes and began to decrease at 60 minutes after treated by HBeAg which implies that CREB participates in the process of HBeAg‐induced macrophage activation. Secondly, to further clarify the relationship between MAPK and CREB, we pre‐treated RAW264.7 with DMSO or inhibitors of ERK, JNK and p38, respectively, then stimulated RAW264.7 with HBeAg for 30 minutes and detected the expression of phosphorylated CREB. It can be seen in Figure 4C, the increased phosphorylated CREB induced by HBeAg was significantly suppressed by ERK and p38 inhibitors. Thus, we conclude that CREB is located downstream of MAPK and is regulated by ERK and p38. In the end, in order to verify whether CREB can regulate the expression of miR‐212‐3p, we performed the q‐PCR and ChIP experiments. On one hand, we pre‐treated RAW264.7 with DMSO or inhibitors of CREB and then stimulated RAW264.7 with HBeAg for 24 hours and detected the expression of miR‐212‐3p by q‐PCR, and it can be seen from Figure 4D, the increased expression of miR‐212‐3p induced by HBeAg is significantly inhibited by CREB inhibitor. On the other hand, to demonstrate that CREB interacts with miR‐212‐3p promoter, a ChIP assay was carried out using anti‐CREB or its corresponding antibody IgG. Figure 4E demonstrates that CREB binding to the miR‐212‐3p proximal promoter in HBeAg‐stimulated group is richer than without stimulation. Taken together, we infer that ERK/CREB signalling pathway promotes the expression of miR‐212‐3p in macrophage induced by HBeAg.

### 3.5 MiR‐212‐3p inhibits HBeAg‐induced inflammatory cytokine production through targeting MAPK1

**FIGURE 5:**
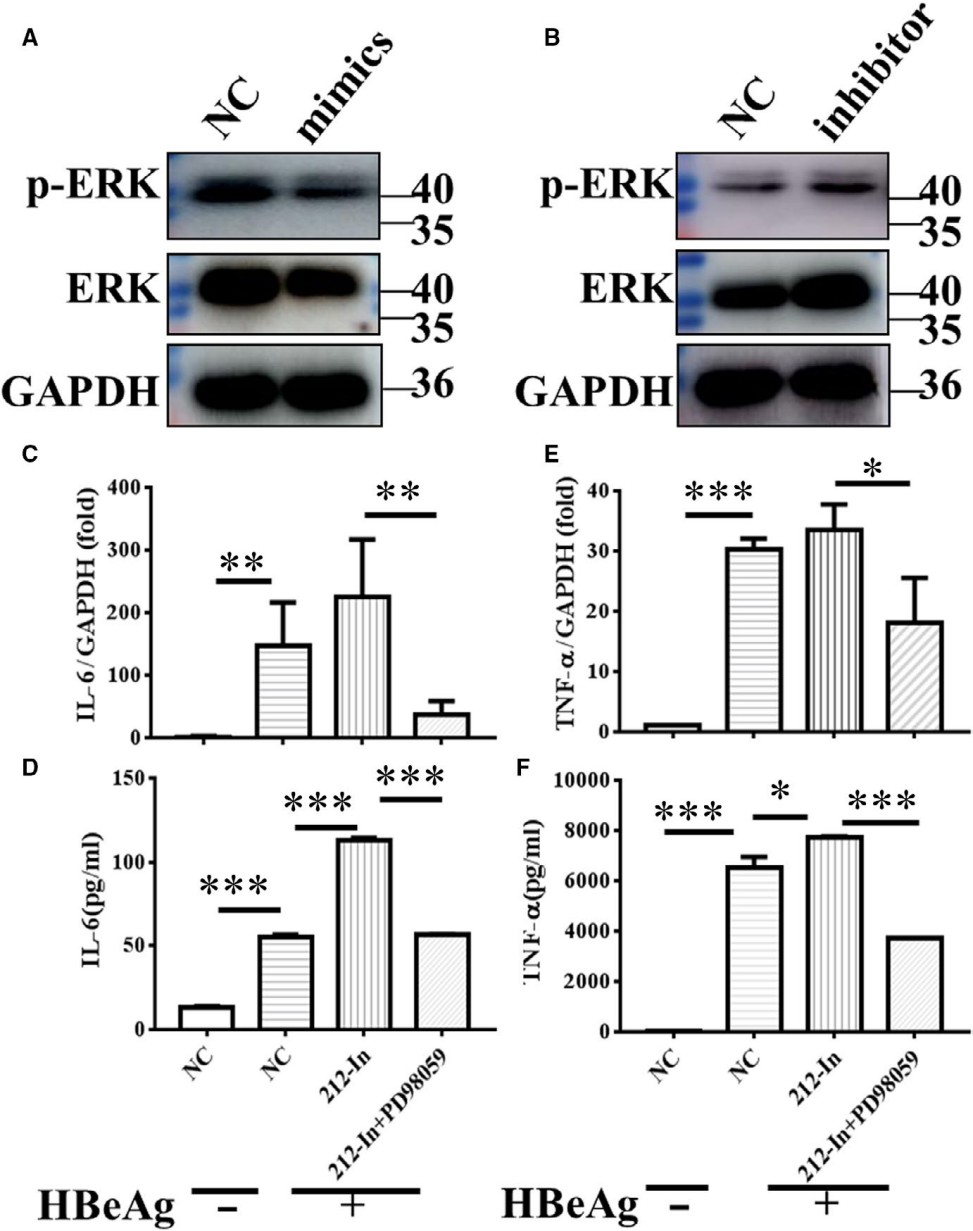
miR‐212‐3p regulated HBeAg‐inducing inflammatory cytokines production via targeting MAPK1. Changes in non‐ phosphorylation and phosphorylation of ERK in RAW264.7 macrophages after transfection with miR‐212‐3p mimics or miR‐212‐3p inhibitor were determined with Western blot analysis (A, B). miR‐212‐3p inhibitor or negative control miRNA were transfected into RAW264.7 for 40 h, then cells were treated with DMSO or inhibitor of ERK (PD98059, 10 μmol/L) for 2 h, and cells were stimulated with HBeAg for 4 h. The expression and production of IL‐6 (C, D) and TNF‐α (E, F) were evaluated with q‐PCR and ELISA. Columns 1 and 2 represent the change of cytokine expression and secretion with or without HBeAg stimulation after transfection of NC, respectively (C‐F). Data are shown by three or four independent experiments (mean ± SD of triplicates in C‐F). *P < 0.05, **P < 0.01, ***P < 0.001

In order to find the target gene of miR‐212‐3p, we performed bioinformatics analysis with starBase (http://starbase.sysu.edu.cn/). MAPK1, also named ERK2, is predicted to be the target of miR‐212‐3p. The binding site of miR‐212‐3p and MAPK1 predicted by different databases is shown in Table 1. Meanwhile, we detected the expression of non‐ phosphorylation and phosphorylation of MAPK1, after transfection with miR‐212‐3p mimics or inhibitor. As can be seen from Figure 5A,B, miR‐212‐3p mimics significantly decreased the expression of total and phosphorylated MAPK1, while miR‐212‐3p inhibitor obviously increased their expression. Next, we used ERK inhibitor PD98059 and pre‐treated RAW264.7 cells transfected with miR‐212‐3p inhibitor and then detected the expression of IL‐6 and TNF‐α after stimulation of HBeAg for 4 hours. As shown in Figure 5C‐F, PD98059 dramatically reversed miR‐212‐3p inhibitor caused the increase of IL‐6 and TNF‐α expression and secretion. All together, these data demonstrated that miR‐212‐3p inhibited the production of inflammatory cytokines in HBeAg‐stimulated macrophages through suppressing the expression of MAPK1.

**TABLE 1:** The predicted miR‐212‐3p binding sites in the 3′‐UTR of MAPK1

Binding site	Class	Alignment	Predicted by database
Chr16:17044980–17044986 [+]	8mer	Target: 5' uuaCUGUGCUCUUGCAUGACUGUUa 3' miRNA: 3'accGGCAC‐‐UGACCU‐CUGACAAu 5'	PITA, miRmap, PicTar
Chr16:17044959–17044987 [+]	8mer	Target: 5' uuaCUGUGCUCUUGCAUGACUGUUa 3' miRNA: 3' accGGCAC‐‐UGACCU‐CUGACAAu 5'	microT
Chr16:17044963–17044987 [+]	8mer	Target: 5' uuaCUGUGCUCUUGCAUGACUGUUa 3' miRNA: 3' accGGCAC‐‐UGACCU‐CUGACAAu 5'	miRanda
Chr16:17044980–17044987 [+]	8mer	Target: 5' uuaCUGUGCUCUUGCAUGACUGUUa 3' miRNA: 3' accGGCAC‐‐UGACCU‐CUGACAAu 5'	TargetScan
Chr16:17043916–17043921 [+]	6mer	Target: 5' auGCACUUAACUGCUUACUGUUg 3' miRNA: 3' acCG‐GCACUGACCUCUGACAAu 5'	PITA, miRmap
Chr16:17043894–17043922 [+]	6mer	Target: 5' auGCACUUAACUGCUUACUGUUg 3' miRNA: 3' acCG‐GCACUGACCUCUGACAAu 5'	microT
Chr16:17044822–17044827 [+]	6mer	Target: 5' aaaaggcuagcaguaACUGUUc 3' miRNA: 3' accggcacugaccucUGACAAu 5'	PITA, miRmap
Chr16:17044800–17044828 [+]	6mer	Target: 5' aaaaggcuagcaguaACUGUUc 3' miRNA: 3' accggcacugaccucUGACAAu 5'	microT
Chr16:17046005–17046011 [+]	7mer‐m8	Target: 5' uuuuuuaaagcacuGACUGUUc 3' miRNA: 3' accggcacugaccuCUGACAAu 5'	PITA, miRmap
Chr16:17045984–17046012 [+]	7mer‐m8	Target: 5' uuuuuuaaagcacuGACUGUUc 3' miRNA: 3' accggcacugaccuCUGACAAu 5'	microT
Chr16:17045991–17046012 [+]	7mer‐m8	Target: 5' uuuuuuaaagcacuGACUGUUc 3' miRNA: 3' accggcacugaccuCUGACAAu 5'	miRanda
Chr16:17046559–17046565 [+]	8mer	Target: 5' acacuugGGCUGGGCAUGACUGUUa 3' miRNA: 3' accggcaCUGACCU‐‐‐CUGACAAu 5'	PITA, miRmap
Chr16:17046538–17046566 [+]	8mer	Target: 5' acacuugGGCUGGGCAUGACUGUUa 3' miRNA: 3' accggcaCUGACCU‐‐‐CUGACAAu 5'	microT
Chr16:17046542–17046566 [+]	8mer	Target: 5' acacuugGGCUGGGCAUGACUGUUa 3' miRNA: 3' accggcaCUGACCU‐‐‐CUGACAAu 5'	miRanda

## 4 DISCUSSION

Hepatitis B virus (HBV) infection is still a major health risk now. Upon exposure to HBV, approximately 90%‐95% of adults will eliminate the virus, while 5%‐10% of them will progress to chronic infection and about 1% of them will develop severe hepatitis B with high fatality rates. The different outcome of infection depends on virus and host factors. For patients, controlling virus infection needs the cooperation of both innate immunity and adaptive immunity. Generally, the innate immunity is immediately started to limit the spread of infection and prompt an efficient adaptive immune response.^20^

Tissue‐resident macrophages serve as the first line of defence against pathogens and are regarded as an important part of innate immunity. Macrophages in liver, also called Kupffer cells, are the largest macrophage population in the human body, which account for about 80% of total human macrophages under physiological conditions.^21^, ^22^ There are multiple pattern recognition receptors (PRRs) including TLR1/2, TLR2/6, TLR3, TLR4, TLR8 and RIGI/MDA5^23^, ^24^ expressed by liver macrophages, which lead to secretion of cytokines (IL‐6, TNF‐α, etc) and chemokines after stimulation.^3^, ^25^, ^26^ Our previous findings demonstrated that HBeAg, but not HBcAg and HBsAg, play a key role in inducing macrophage activation and exacerbate liver injury by increasing the production of inflammatory cytokines.^6^

MiR‐212, located on chromosome 17q13.3, was first revealed to play an essential role in different aspects of neurons including development, maturation, morphogenesis and function. The abnormal expression of miR‐212 may cause a series of neurodegenerative diseases, such as Alzheimer's disease, epilepsy, tauopathies, schizophrenia and so on. Apart from nervous system disease, miR‐212 was also found to be abnormally expressed in cancer and play an important role in tumour immunity.^27^ For example, the down‐regulated miR‐212‐3p was found to regulate prostate cancer (PCa) development through promoting the secretion of inflammatory cytokines via NF‐κB pathway.^28^ Another research showed that the SNP rs1599795 in CD80 3′‐UTR, through disrupting the regulatory role of miR‐212‐3p in CD80 expression, contributed to tumorigenesis of gastric cancer.^29^ In pancreatic cancer, miR‐212‐3p was revealed to induce immunologic tolerance of dendritic cells.^30^ Recently, miR‐212‐3p was demonstrated to play a role in the inflammatory response of macrophages. Chen et al found that miR‐212‐3p suppressed LPS‐induced inflammatory cytokine production in macrophage,^31^ while little is known about the relationship between miR‐212‐3p and macrophages in viral infectious disease, especially HBV infection. This study is the first to demonstrate that miR‐212‐3p can regulate the production of inflammatory cytokines in HBeAg‐induced macrophages.

The role of macrophages in liver has attracted a lot of attention recently, and they were found to play a crucial part in liver inflammation/ tolerance and injury associated with uncontrolled inflammation.^32^ In the case of HBV infection, hepatic macrophages can contribute not only to inhibit virus replication but also to activate antiviral immunity.^33^ Our findings indicated that miR‐212‐3p is up‐regulated in HBeAg‐stimulated macrophages of human and mouse. Overexpression of miR‐212‐3p decreased the production of IL‐6 and TNF‐α at both mRNA and protein level in HBeAg‐induced macrophage, whereas knockdown of miR‐212‐3p had the opposite effect.

MAP kinases (ERK, JNK, p38) play a key role in the modulation of cell growth and differentiation and regulate inflammatory and immune responses.^34^, ^35^ According to Park JS, the p38 MAP kinase plays a primary role in inducing pro‐inflammatory cytokine expression in LPS‐induced neutrophils.^36^ In our present study, we found that ERK, JNK and p38 were all activated in HBeAg‐induced macrophages. In addition, with the use of ERK, JNK and p38 inhibitors, respectively, the production of IL‐6 and TNF‐α at protein level after being treated with HBeAg was all significantly suppressed. Apart from regulating pro‐inflammatory cytokine expression, can MAPK regulate the expression of miR‐212‐3p? It was reported that ERK1/2 regulated the expression of pri‐miR‐212/132 transcription in BDNF‐stimulated primary cortical neurons; in addition, they also found that SB203580, a p38 MAPK inhibitor, led to an increase in pri‐miR‐212/132 transcription.^37^ In this study, we identified that only ERK inhibitor blocked the expression of miR‐212‐3p, whereas p38 and JNK inhibitors slightly increased its expression. So, we concluded that ERK regulated the expression of miR‐212‐3p, and we speculated that JNK and p38 may regulate the expression of miR‐212‐3p by other adaptor. CREB, as an important transcription factor, was activated in HBeAg‐induced macrophages, which implied the important role of CREB in this process. Meanwhile, ERK is reported to regulate transcription not only by the direct phosphorylation of specific transcription factors but also by the activation of downstream kinases, such as RSK and MSK, which may in turn phosphorylate chromatin and transcription factors.^38^, ^39^ For example, MSKs have been revealed to phosphorylate CREB on Ser^133^ downstream of ERK.^40^ In our study, we confirmed that both ERK and p38 signalling could regulate the phosphorylation of CREB. In order to prove that CREB can regulate the expression of miR‐212‐3p, we showed that the inhibitor of CREB (KG‐501) could block the expression of miR‐212‐3p and the ChIP assay indicated the interaction of CREB and miR‐212‐3p promoter. KG‐501, as a CREB functional inhibitor, was shown by multiple studies to specifically inhibit the transcription activity of phosphorylated CREB.^41^, ^42^ Therefore, we concluded that miR‐212‐3p was regulated by ERK/CREB signal pathway.

**FIGURE 6:**
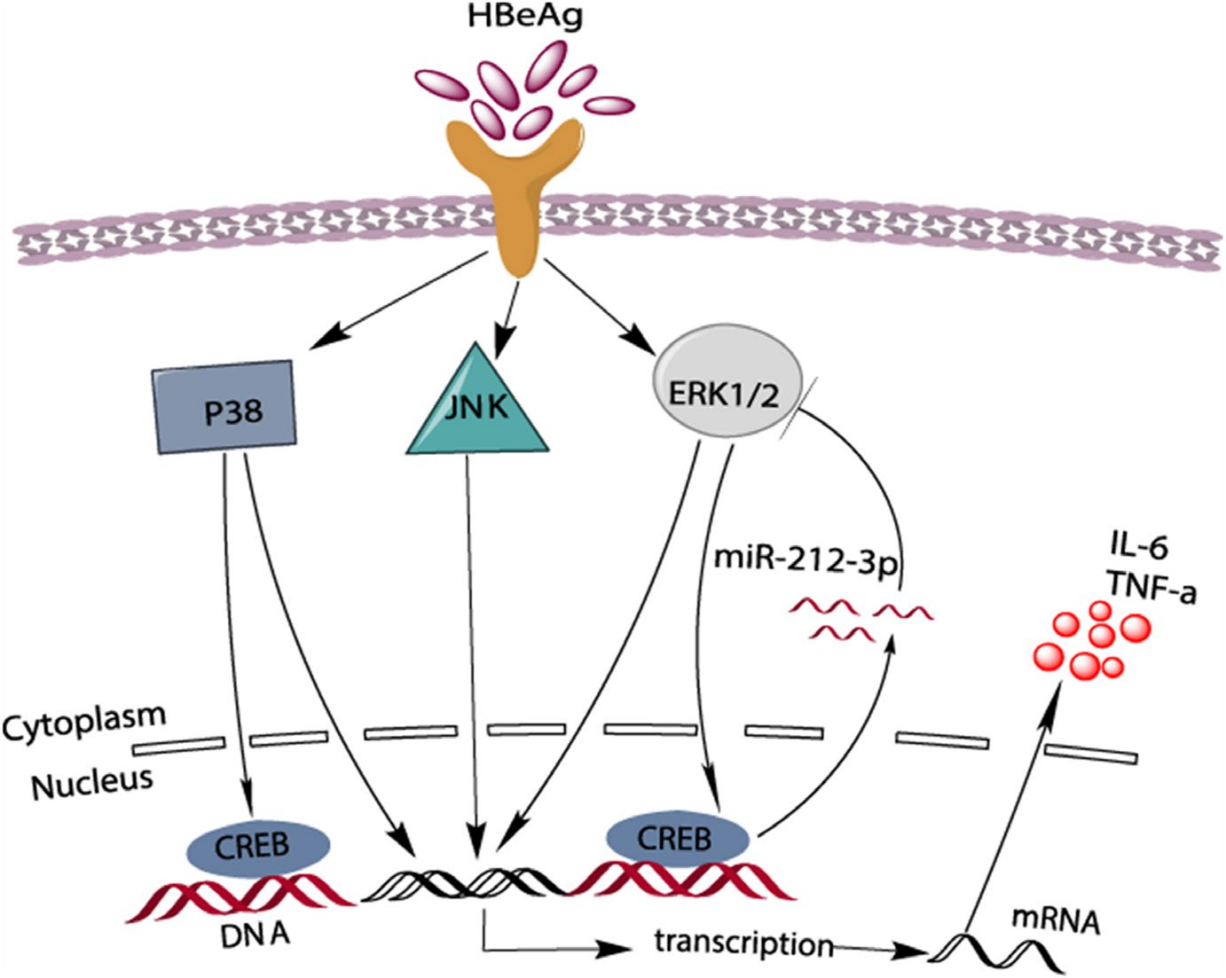
HBeAg from HBV stimulated macrophages and activated MAPK pathway. This eventually led to gene transcription and production of IL‐6 and TNF‐α. Both ERK and p38 may activate CREB, while ERK/CREB signalling led to the production of miR‐212‐3p. miR‐212‐3p, in turn, inhibited the production of cytokines by targeting MAPK1 (A)

MiR‐212‐3p was reported to exert tumour‐suppressive roles by targeting MAPK1, also named ERK2, in prostate cancer.^43^ In the present study, we found that ERK1/2 can regulate the production of cytokines as well as the expression of miR‐212‐3p in macrophages. Interestingly, we also verified that miR‐212‐3p can inhibit macrophages inflammatory cytokines production by suppressing MAPK1, which formed a negative feedback loop to modulate the inflammatory process in macrophages Figure 6A.

In conclusion, our results demonstrated that HBeAg promotes the expression of miR‐212‐3p in macrophages. In addition, we testified that ERK/CREB signalling pathway regulates the expression of miR‐212‐3p. Moreover, miR‐212‐3p negatively modulated inflammatory cytokine production in HBeAg‐induced macrophage activation via targeting MAPK1. Our findings further contributed to our understanding of the mechanism of inflammation regulation by miR‐212‐3p in HBeAg‐induced macrophage activation. That means we may control inflammation progression and outcome by modulating miR‐212‐3p during HBV infection.

## CONFLICTS OF INTEREST

The authors declare no conflict of interest.

## AUTHOR CONTRIBUTION

**Wenjun Chen:** Data curation (lead); investigation (lead); methodology (lead); writing‐original draft (lead). **Hongjun Bian:** Data curation (supporting); investigation (lead); methodology (supporting); writing‐original draft (lead). **Xiaoyu Xie:** Investigation (lead); methodology (lead). **Xia Yang:** Data curation (supporting); methodology (supporting). **Benjun Bi:** Formal analysis (supporting); methodology (supporting). **Chunliu Li:** Formal analysis (supporting); investigation (supporting). **Yuejuan Zhang:** Investigation (supporting); methodology (supporting). **Qiang Zhu:** Data curation (supporting); funding acquisition (supporting); project administration (supporting); writing – review and editing (supporting). **Jing Song:** Project administration (supporting). **Chengyong Qin:** Conceptualization (supporting); Data Curation (Supporting); Funding Acquisition (Supporting); Project Administration (Supporting); Supervision (Supporting); Writing‐Original Draft (supporting); writing – review and editing (supporting). **Jianni Qi:** Conceptualization (lead); data curation (lead); formal analysis (lead); funding acquisition (lead); investigation (lead); project administration (lead); supervision (lead); writing‐original draft (lead); writing – review and editing (lead).

## Data Availability

The data that support the findings of this study are available from the corresponding author upon reasonable request.
